# Effect of beinaglutide combined with metformin versus aspart 30 with metformin on metabolic profiles and antidrug antibodies in patients with type 2 diabetes: a randomized clinical trial

**DOI:** 10.3389/fendo.2023.1267503

**Published:** 2023-12-06

**Authors:** Chen-Yu Han, Jia-Ping Lu, Xiao-Mei Ye, Hai-Ying Jin, Wei-Wei Xu, Ping Wang, Min Zhang

**Affiliations:** Department of Endocrinology, Qingpu Hospital Affiliated to Fudan University, Shanghai, China

**Keywords:** type 2 diabetes, beinaglutide, anti-drug antibodies, non-fasting triglyceride, cardiovascular disease

## Abstract

**Objective:**

This prospective study aimed to evaluate the effect of beinaglutide combined with metformin versus aspart 30 with metformin on metabolic profiles and antidrug antibodies (ADAs) in patients with type 2 diabetes (T2D).

**Methods:**

A total of 134 eligible participants were randomly assigned to the test group and the control group. Patients in the test group were treated with beinaglutide and metformin, whereas patients in the control group were randomly treated with aspart 30 and metformin, with a follow-up period of 6 months. The metabolic profiles and ADAs over 6 months were evaluated.

**Results:**

After 6 months, 101 (75.37%) patients completed the study. Compared with the control group, the beinaglutide group had significant reductions in 2-h postprandial blood glucose (2hBG) and low blood glucose index (LBGI). Glycated hemoglobin (HbA1c) decreased in both groups relative to baseline. In the test group, one had treatment-emergent beinaglutide ADAs. Significant reductions in triglycerides (TG), non-fasting TG, weight, waist circumference (WC), and body mass index (BMI) were observed. The values of insulin sensitivity index (HOMA-IR) were decreased to a statistically higher degree with beinaglutide treatment.

**Conclusion:**

Beinaglutide reduces metabolic dysfunction, LBGI, and weight in patients of T2D with a low risk of ADAs. Beinaglutide may offer the potential for a disease-modifying intervention in cardiovascular disease (CVD).

**Clinical trial registration:**

www.chictr.org.cn, identifier ChiCTR2200061003.

## Introduction

Type 2 diabetes (T2D) epidemic is a major health concern, and obesity is contributing to the increase in T2D prevalence (1). T2D is currently considered a systemic disease in which there is dysfunction in multiple organs and tissues (2). Disturbed glucose levels due to obesity-related insulin resistance or release of inflammatory factors cause endothelial and smooth muscle cell dysfunction (3). Thus, microvascular and macrovascular complications are common complications of T2D and the latter remains the most common cause of death (4). People with diabetes have a 1.6 to 2.6 times increased risk of cardiovascular disease (CVD) compared with non-diabetics (5). Obesity and dyslipidemia are important contributors to the increased risk of CVD. Recently, triglyceride (TG) levels have become a predictor and therapeutic target for reducing CVD (6). In clinical practice, serum TG values are usually measured after an 8–12-h fast to avoid the effects of diet. However, most people are not fasting except for a few hours in the early morning. Therefore, fasting serum TG levels may not reflect average daily serum TG levels, which may have a greater impact on atherosclerosis (7). Therefore, the non-fasting period reflects the true atherosclerotic load better than the fasting period (8).

Glucagon-like peptide-1 receptor agonists (GLP-1RAs) are a new emerging drug class in treatment of T2D that act on several targets including the pancreatic β-cell, liver, kidney, brain, and CV system (9). In general, GLP-1 RAs reduce body weight, lower blood pressure, and positively affect the lipid profile (10). Other pleiotropic effects could involve protection against CVD since GLP-1 receptors have been found in the heart and endothelium (11). Different homologies to human GLP-1 (7–36) among GLP-1 RAs may result in various actions, efficacy, and tolerability of these medications in the administration (12). Beinaglutide is one of the GLP-1 RAs that have 100% homology to human GLP-1 approved in China for the treatment of patients with T2D (13). Previous studies have shown that patients with T2D who were treated with beinaglutide for 3 months have a mean reduction in body weight of 10.05 kg and improved glycemic control (14). Currently, there is still limited evidence for the incidence of antidrug antibodies (ADAs) and the effect on non-fasting TG levels of beinaglutide. The aim of this study was to evaluate the effect of beinaglutide combined with metformin versus aspart 30 with metformin on metabolic profiles including glucose and lipid profile and ADAs of beinaglutide in patients with T2D.

## Methods

### Study design and participants

A prospective randomized controlled study was conducted from 14/02/2020 to 26/08.2022 in Qingpu Hospital Affiliated to Fudan University. Ethical approval was obtained by the Ethical Committee of Qingpu Hospital Affiliated to Fudan University (ID number: IEC-C-007-A08-V.03), and all participants provided a written informed consent. The study was also registered on www.chictr.org.cn (ID number ChiCTR2200061003).

Sample size was determined based on glycated hemoglobin (HbA1c), a key variable from previous studies (15). Considering a = 0.05 and statistical efficacy 1 − b = 0.8, the sample size was calculated as 47 cases in each group. Of 490 prescreened T2D patients, 449 met the eligibility criteria. Taking into account the 30% dropout rate, a total of 134 participants (67 per group) were included in the study. All participants diagnosed with T2D according to World Health Organization (WHO) 1999 criteria (16) underwent monotherapy with metformin but with poor metabolic control (7%≤ HbA1c ≤10%). Inclusion criteria included the following: subjects willing and able to comply with the requirements of the trial program and agree to sign the informed consent form; subjects aged 18–65 on the date of signing the informed consent form (including the threshold); at least 8 weeks of stable treatment with metformin alone (daily dose ≥1,000 mg) prior to screening; body mass index (BMI) of 22.0–40.0 kg/m^2^ (both extremes) at screening and change in body weight (difference between maximum and minimum body weight in 3 months) of not more than 5 kg in the 3 months prior to screening. The following were the exclusion criteria: treated with GLP-1RAs previously; acute complications of diabetes such as diabetic ketoacidosis (DKA) or hyperosmotic hyperglycemia syndrome (HHS) that occurred in the past 6 months; glucocorticoid treatment (oral or intravenous) that lasted for more than 7 days within 6 months; have a history of idiopathic pancreatitis, chronic pancreatitis, or gastrointestinal diseases; moderate to severe renal insufficiency or end-stage renal disease; there were significant cardiovascular and cerebrovascular events within 3 months; pregnant and lactating women.

### Intervention

Participants who met the inclusion criteria were randomly divided into two groups at a 1:1 ratio. Patients in the test group (n = 67) were randomly treated with beinaglutide and metformin, whereas patients in the control group (n = 67) were randomly treated with aspart 30 and metformin, with a follow-up period of 6 months. The recommended dosage of beinaglutide is 0.1mg three times daily (tid) by subcutaneous injection in the upper arm, thigh, or abdomen. The dosage may be increased to 0.2 mg tid if the glycemic response is inadequate (17). The dosage of aspart 30 is determined by the endocrinologist according to the patient’s blood glucose (BG).

### Outcomes

The primary objective of this study was to demonstrate the superiority of beinaglutide versus aspart 30 in reducing blood glucose and the low blood glucose index (LBGI) levels after 6 months. At the same time, the incidence of ADAs was used to assess the safety of beinaglutide. The secondary efficacy endpoints were lipid profile, especially non-fasting TG levels. Other endpoints were weight, waist circumference (WC), BMI, subcutaneous fat, and insulin sensitivity index (HOMA-IR).

### Follow-up and outcome measurements

All participants were required to undergo anthropometric and laboratory examinations at baseline and after the intervention. Height (in centimeters) was measured using a wall-mounted height-measuring device, barefoot. Weight was measured with light clothing and no shoes, and the same electronic scale is used before and after the intervention. The previous calibration error may be ±100 g. BMI is calculated in kg/m^2^. WC is measured while standing, with the tape measure placed above the umbilical cord, against the skin.

### Blood samples

Venous blood was drawn at 5:30 am after fasting for 12 h. Blood samples were sent to the laboratory within an hour. The serum levels of fasting blood glucose (FBG), total cholesterol (TC), TG, low density lipoprotein (LDL) cholesterol, and high density lipoprotein cholesterol (HDL) were measured using the standard enzymatic procedure (Hitachi LABOSPECT 008 AS, Japan).

Non-fasting TG was taken 120 min after eating 100 g of standard carbohydrate provided by China Foods Limited (Beijing, China) (18). Blood was used with K2EDTA anticoagulant for HbA1c and determined by high-performance liquid chromatography (HPLC) (Tosoh HLC-723G11, Japan).

The commercial Roche test on the Cobas 8000/801 automatic analyzer (Roche Diagnostics, Mannheim, Germany) was used to measure fasting insulin (FI) by electrochemiluminescence (ECL). HOMA-IR was calculated by the formula HOMA-IR = FBG (mmol/L) × FI (mIU/L)/22.5 (19).

LBGI was used to evaluate the frequency and degree of hypoglycemia based on the mathematical processing of BG measurements. The specific calculation methods were as follows:

1) The BG value was transformed:


$$X_{i}=1.794\times \left\{{\left[\left(\ln G_{i}\right)\right]}^{1.026}-1.861\right\}$$

*Xi* is the converted BG; G is the measured BG.

2) The risk value for BG was calculated according to *Xi*:


$$\text{LBGI}=\frac{1}{N}\sum_{t=0}^{N}\text{rl}\left({\text{x}}_{\text{i}}\right)$$

N is the total number of blood glucose measurements; rl is the risk of hypoglycemia (*Xi*<0) (20).

ADAs of beinaglutide were measured by the electrochemiluminescence method, which is based on beinaglutide labeled with biotin and metal ruthenium, using the classic bridging principle to detect ADAs against beinaglutide in human serum. The ruthenium in the complex “biotin-labeled beinaglutide–ADAs–ruthenium-labeled beinaglutide” excites a light signal value at 620 nm and is captured by an MSD (Meso Scale Discovery) instrument. The captured signal intensity is proportional to the titer of ADAs.

### Statistical analysis

Baseline and post-intervention data were summarized as mean and standard deviation (SD) for normal variables, median and quartile ranges for abnormal variables, and number and percentage for categorical variables. Baseline and 6-month follow-up characteristics of each group were compared using an independent two-sample t test or chi-square test. Paired t test was used to compare the differences before and after the same intervention. The missing values were interpolated with the mean. Double-tailed P value<0.05 was considered statistically significant. SPSS Statistics 25 (IBM) software was used for statistical analysis.

## Results

**Figure 1:**
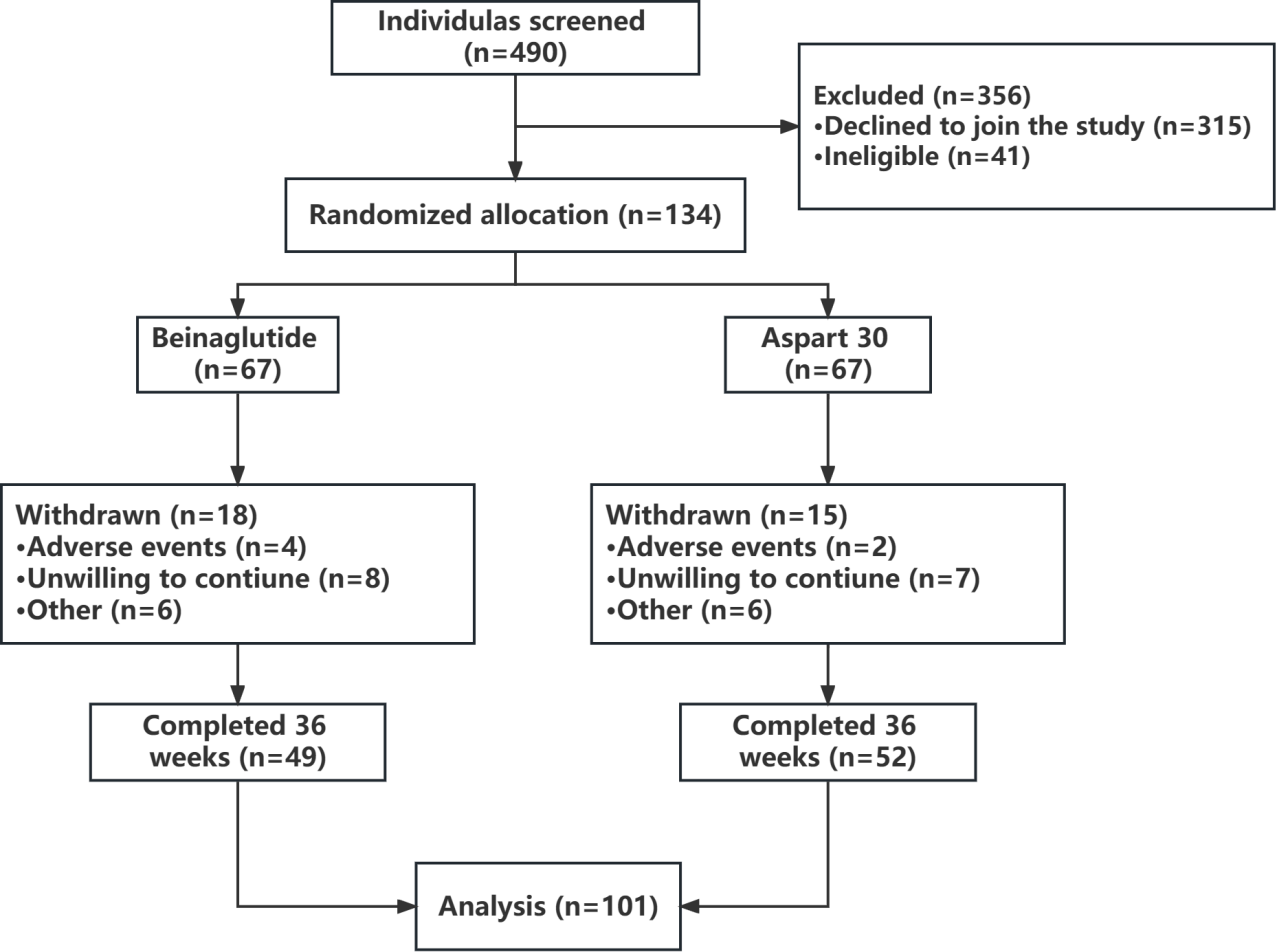
Study design and flowchart.

**Table 1** shows the baseline demographic characteristics of the study population. There were no significant differences in clinical and biochemical indexes among all groups. Of the 490 prescreened patients, 134 agreed to participate in the study. Of the 134 participants with T2D starting on treatment, 101 patients completed the study (**Figure 1**).

**Table 1:** Patients’ characteristics on baseline.

Characteristics	Beinaglutide (n=67)	Control (n=67)	*p* value*
Age (years)	52.1 ± 9.2	51.8 ± 8.3	0.68
FBG (mmol/L)	7.47 ± 1.77	7.86 ± 2.16	0.25
2hBG (mmol/L)	12.94 ± 2.23	13.26 ± 2.63	0.92
HbA1c%	9.45 (7.70,11.22)	9.60 (8.10,11.91)	0.29
LBGI	2.62 ± 1.81	2.71 ± 1.20	0.54
Weight (kg)	84.34 ± 13.86	83.12 ± 16.09	0.64
BMI (kg/m^2^)	27.08 ± 3.64	26.87 ± 2.94	0.71
WC (cm)	98.15 ± 9.67	97.44 ± 8.29	0.65
SFA (cm^2^)	175.96 ± 37.47	171.86 ± 39.49	0.25
ALT (U/L)	26.67 (19.96,37.15)	23.00 (17.00,37.12)	0.22
AST (U/L)	26.57 (19.00,35.00)	23.00 (17.00,33.00)	0.24
Cr (mmol/L)	70.82 ± 21.43	69.16 ± 20.68	0.65
UA (mmol/L)	364.96 ± 87.96	357.29 ± 91.75	0.26
Non-fasting TG (mmol/L)	2.74 ± 5.96	2.68 ± 5.61	0.96
TC (mmol/L)	4.96 (4.33,6.10)	4.96 (4.10,6.47)	0.71
HDL-C (mmol/L)	1.00 ± 0.24	1.04 ± 0.22	0.26
LDL-C (mmol/L)	2.93 ± 0.85	2.85 ± 0.84	0.61
TG(mmol/L)	2.12 (1.23,2.91)	2.24 (1.23,3.08)	0.75
ApoB (mg/dL)	1.01 ± 0.41	1.02 ± 0.36	0.89
FFA (nmol/L)	0.42 ± 0.20	0.39 ± 0.17	0.43
HOMA-IR	2.40 (1.16,3.23)	2.41 (1.19,3.19)	0.90

*p<0.05.

Compared with the control group, the beinaglutide group had significant reductions in 2hBG (p< 0.01) and LBGI (p = 0.02) (**Table 2**). HbA1c decreased in both groups relative to baseline (p< 0.01) (**Table 2**). There was no significant difference in HbA1c levels between groups (p = 0.86). In the beinaglutide group, one had treatment-emergent beinaglutide ADAs, exhibiting low titers. For the lipid profile, beinaglutide significantly reduced FTG and non-fasting triglyceride (TG) levels after 6 months of supplementation (p<0.01) (**Table 2**). At the same time, significant reductions in weight, WC, hip, and BMI were observed (p<0.05) (**Table 2**). The values of HOMA-IR were decreased to a statistically higher degree with beinaglutide treatment (p<0.01) (**Table 2**).

**Table 2:** Comparison of the differences between groups.

	Beinaglutide		P value	Control		*P* value	*P* value
	Baseline	6-month		Baseline	6-month		
FBG (mmol/L)	7.47 ± 1.77	6.67 ± 1.90	0.02	7.86 ± 2.16	6.90 ± 2.17	<0.01	<0.01
2hBG (mmol/L)	12.94 ± 2.23	8.02 ± 1.72	<0.01	13.26 ± 2.63	10.34 ± 2.99	0.38	<0.01
HbA1c%	9.45 (7.70,11.22)	6.87 (4.90,8.25)	<0.01	9.60 (8.10,11.91)	6.87 (5.29,8.79)	<0.01	0.86
LBGI	2.62 ± 1.81	2.12 ± 0.94	0.03	2.71 ± 1.20	2.52 ± 1.16	0.22	<0.01
Weight (kg)	84.34 ± 13.86	79.30 ± 12.92	0.039	83.12 ± 16.09	85.39 ± 15.94	0.65	<0.01
BMI (kg/m^2^)	27.08 ± 3.64	26.08 ± 2.89	0.029	26.87 ± 2.94	27.40 ± 2.87	0.56	<0.01
WC (cm)	98.15 ± 9.67	93.62 ± 9.64	0.086	97.44 ± 8.29	98.38 ± 8.00	0.86	<0.01
SFA (cm^2^)	175.96 ± 37.47	171.34 ± 40.30	0.63	171.86 ± 39.49	172.64 ± 41.66	0.99	0.07
LMI (kg/m^2^)	19.08 ± 2.87	20.08 ± 1.79	0.92	19.87 ± 3.56	1840 ± 2.87	0.58	0.77
ALT (U/L)	26.67 (19.96,37.15)	25.46 (17.79,37.63)	0.81	23.00 (17.00,37.12)	23.03 (16.24,34.71)	0.80	0.52
AST (U/L)	26.57 (19.00,35.00)	27.37 (17.73,35.17)	0.77	23.00 (17.00,33.00)	22.89 (17.08,33.33)	0.73	0.64
Cr (mmol/L)	70.82 ± 21.43	60.47 ± 22.36	0.092	69.16 ± 20.68	67.53 ± 21.38	0.35	0.07
UA (mmol/L)	364.96 ± 87.96	357.61 ± 81.49	0.55	357.29 ± 91.75	359.34 ± 82.29	0.76	0.28
Non-fasting TG (mmol/L)	2.74 ± 5.96	1.67 ± 3.50	<0.01	2.68 ± 5.61	2.64 ± 5.27	0.84	<0.01
TC (mmol/L)	4.96 (4.33,6.10)	4.41 (3.79,5.67)	0.08	4.96 (4.10,6.47)	4.45 (3.56,5.72)	0.13	0.78
HDL-C (mmol/L)	1.00 ± 0.24	1.17 ± 0.23	<0.01	1.04 ± 0.22	1.23 ± 0.21	<0.01	0.88
LDL-C (mmol/L)	2.93 ± 0.85	2.58 ± 0.78	0.05	2.85 ± 0.84	2.53 ± 0.76	0.07	0.28
TG (mmol/L)	2.12 (1.23,2.91)	1.61 (0.98,2.61)	0.08	2.24 (1.23,3.08)	2.16 (1.27,2.91)	0.72	<0.01
ApoB (mg/dL)	1.01 ± 0.41	0.96 ± 0.38	0.62	1.02 ± 0.36	1.02 ± 0.36	0.99	0.34
FFA (nmol/L)	0.42 ± 0.20	0.40 ± 0.20	0.61	0.39 ± 0.17	0.38 ± 0.16	0.96	0.57
HOMA-IR	2.40 (1.16,3.23)	2.03 (1.25,3.49)	0.04	2.41 (1.19,3.19)	2.95 (1.11,2.95)	0.03	<0.01

## Discussion

This study aimed to evaluate the efficacy and safety of beinaglutide in treatment of T2D. After 6 months, HbA1c decreased in both groups relative to baseline and the beinaglutide group had significant reductions in 2hBG compared with the control group. The LBGI of the test group was significantly lower than that of the control group, and no patient experienced hypoglycemia in the test group, suggesting that beinaglutide offered a treatment option that improved glycemic control with a low risk of hypoglycemia. Similar results were obtained in the study by Gao, Lijun et al. (13). Beinaglutide is a short-acting recombinant human GLP-1 (21). GLP-1, as a type of incretins, secreted from L cells in the distal ileum and colon within minutes of oral glucose load (22). Islet B cells contain GLP-1 receptors that, when activated by GLP-1, enhance insulin secretion. Incretins are responsible for around 50% to 70% of total insulin release (23). This clarifies why, around 50 years ago, it was observed that oral glucose intake leads to higher insulin release compared with an equal amount of intravenous glucose (23).

**Figure 2:**
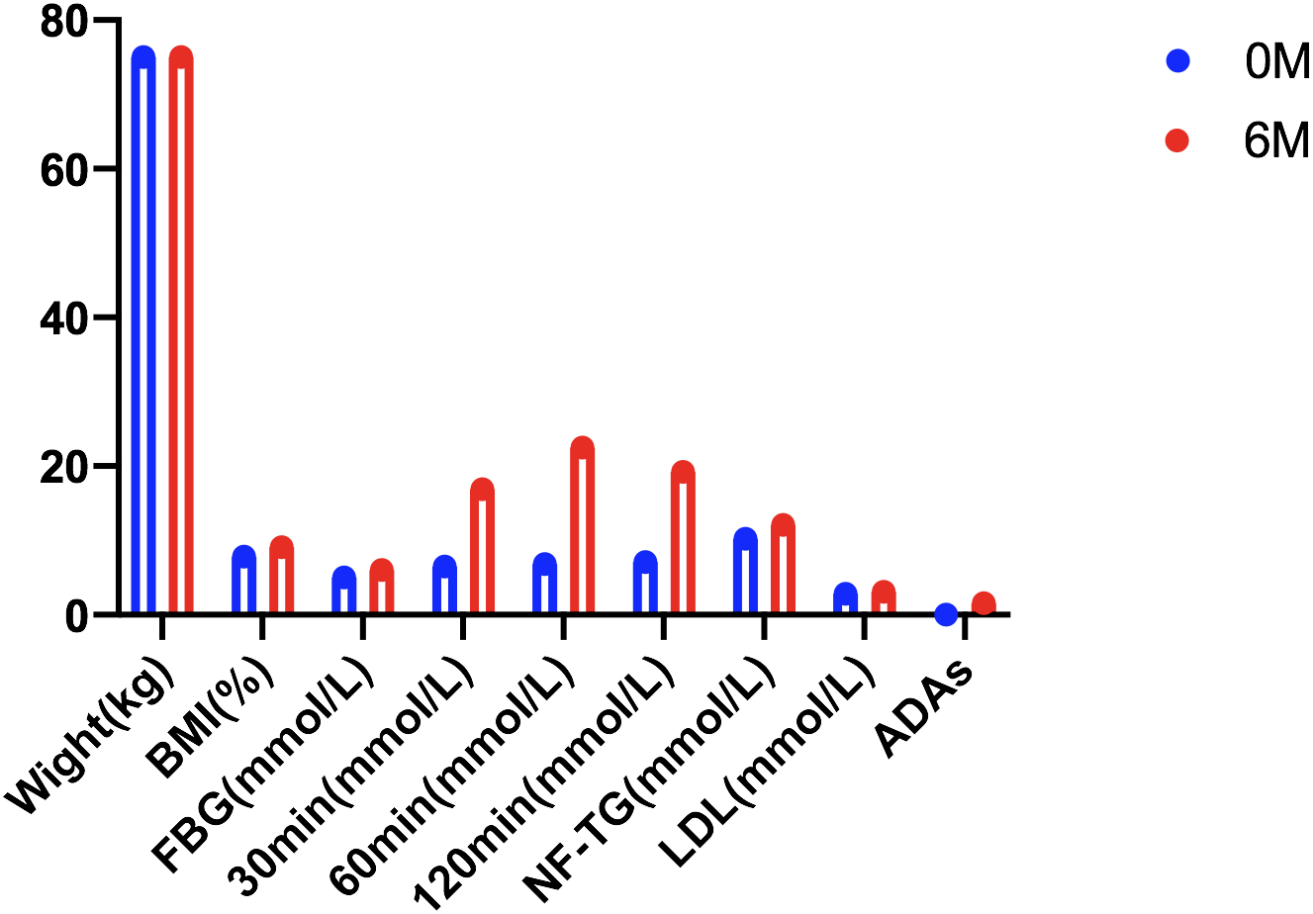
Clinical indicators of the patient with treatment-emergent beinaglutide ADAs.

Beinaglutide is a recombinant human GLP-1 analog with an identical amino acid sequence to native human GLP-1 (24). Zhang et al. postulated that beinaglutide may facilitate its beneficial effects by stimulating GLP-1R-dependent 3′5′-cyclic adenosine monophosphate (cAMP) generation in HEK 293 cells (12). This in turn augments insulin secretion in mice in response to glucose. Furthermore, beinaglutide has a half-life of approximately 5 min (25), akin to the half-life of endogenous GLP-1, which ranges from approximately 1.5 to 5 min. Consequently, compared with other long-acting GLP-1 receptor agonists, beinaglutide has a shorter onset time, rapidly reduces blood glucose levels, and more effectively controls postprandial blood glucose. At the 6-month follow-up in this study, the beinaglutide group showed a significant improvement in their 2hBG levels when compared with the control group. Additionally, the level of HbA1c decreased significantly from the initial value, and this reduction was as effective as that achieved with aspart 30. However, beinaglutide carries a reduced hypoglycemia risk. At the 6-month follow-up, the beinaglutide group exhibited significantly lower LBGI levels compared with the control group, which can be attributed to beinaglutide’s glucose-dependent hypoglycemic mechanism (26). In this study, antidrug antibodies to beinaglutide, a compound exhibiting 100% homology with human endogenous GLP-1 and minimal immunogenicity, were detected for the first time. Out of the 49 patients in the beinaglutide group, one patient generated ADAs during the 6 months of treatment. The patient with treatment-emergent beinaglutide ADAs showed a significant increase in FBG and HbA1c compared with baseline at the 6-month follow-up, and no weight loss was observed (**Figure 2**), indirectly confirming the production of ADAs. This patient was not observed to have a progressive increase in titers in 6 months. In summary, beinaglutide exhibits low hypoglycemia risk, minimal immunogenicity, and strong safety while effectively lowering blood glucose levels.

Postprandial elevated lipid levels are a significant aspect of dyslipidemia in patients with T2DM and are linked to atherosclerosis (5). Animal experiments have shown that beinaglutide can counteract weight gain from a high-fat diet, promote fat storage in adipose tissue, and improve the lipid profile associated with obesity (24). This study examined the effect of beinaglutide on non-fasting TG in T2D subjects for the first time, with a prominent decrease after use. In the fasting state, only hepatogenic lipoproteins are present in plasma, whereas in the non-fasting state, enterogenic lipoproteins coexist with hepatogenic lipoproteins in plasma (27). The development of atherosclerosis begins with damage to the endothelium of the artery, after which lipoproteins enter the intima (28). The monocytes are then activated and transformed into foam cells in the lining of the artery; then, the foam cells collect cholesterol and triglycerides from the blood (29). The exact mechanism through which GLP-1 and GLP-1 RAs influence postprandial lipid and lipoprotein metabolism is not fully elucidated at this time. However, it is commonly associated with enhanced clearance of intestinal lipoproteins. Beinaglutide, a recombinant human GLP-1, notably slows down the emptying of the stomach (30), a trait shared to some extent by the GLP-1 RAs used in clinical practice. This delayed gastric emptying results in a postponement of the passage of high-fat foods into the small intestine. GLP-1 suppresses gastric lipase secretion and intestinal movement (31). The findings of this study suggest that beinaglutide is effective in reducing body weight, alleviating insulin resistance, and improving insulin sensitivity. Insulin is a strong stimulator of lipoprotein lipase, speeding up the removal of triglyceride-rich particles after meals (32). In conclusion, it is plausible to suggest that various mechanisms, including those mentioned, could contribute to the impact of beinaglutide and other GLP-1 RAs on postprandial lipid metabolism. This study demonstrated that beinaglutide can influence postprandial TG in an “anti-atherogenic” fashion in patients with T2D, which related to atherosclerosis and CVD outcomes (5).

Our study had some limitations. Firstly, it was a randomized but not a placebo controlled trial. Secondly, it is a non-multicenter, non-double-blind study with a short follow-up time. Thirdly, only the ADAs of beinaglutide in the test group were tested, and the number of subjects was relatively small. All of this might have significantly affected the quality of the data.

## Conclusion

Our study has indicated that beinaglutide improved glycemic control with a low risk of hypoglycemia and low titers of beinaglutide ADAs. In addition, beinaglutide reduced non-fasting TG, slowing atherogenesis and the progression of CVD *via* direct and indirect mechanisms, which may be a benefit beyond its pleiotropic effects.

## Data availability statement

The raw data supporting the conclusions of this article will be made available by the authors, without undue reservation.

## Ethics statement

The studies involving humans were approved by Ethical Committee of Qingpu Branch of Zhongshan Hospital Affiliated to Fudan University. The studies were conducted in accordance with the local legislation and institutional requirements. The participants provided their written informed consent to participate in this study.

## Author contributions

C-YH: Conceptualization, Writing – review & editing, Investigation, Writing – original draft. J-PL: Writing – review & editing, Data curation, Methodology. X-MY: Writing – review & editing, Validation. H-YJ: Writing – review & editing, Formal analysis. W-WX: Writing – review & editing, Resources. PW: Resources, Writing – review & editing. MZ: Writing – review & editing, Conceptualization.
